# Integrated Chemical and Biological Profiling of Selected Wild *Allium* Species from Kyrgyz Alatau

**DOI:** 10.3390/molecules31183266

**Published:** 2026-09-15

**Authors:** Nadezhda Gemejiyeva, Dinara Myrzabekova, Raya Arysbaeva, Madina Ramazanova, Kamshat Shormakova, Galina Ponomareva, Anastassiya Gadetskaya, Dmitriy Berillo, Zhanat Karzhaubekova

**Affiliations:** 1 Institute of Botany and Phytointroduction, Almaty 050040, Kazakhstan; 2 Almaty Branch of RSE REM “Infracos”, Almaty 050000, Kazakhstan; 3 Department of Science and Technological Development, Manash Kozybayev North Kazakhstan University (NKU), Petropavlovsk 150000, Kazakhstan

**Keywords:** *Allium* species, elemental composition, GC-MS and LC-MS profiles

## Abstract

Wild *Allium* L. species are important sources of mineral elements and structurally diverse primary and specialized metabolites, yet comparative information integrating their elemental composition, lipophilic constituents, polar metabolites, and biological activity remains limited. This study compared the chemical diversity of four wild *Allium* L. taxa—*A. atrosanguineum* var. *fedschenkoanum*, *A. trachyscordum*, *A. turkestanicum*, and *A. suworowii*—collected from different environments. Elemental composition was measured by atomic absorption spectrometry in samples collected at naturally occurring phenological stages. Chloroform-soluble metabolites were analyzed by gas chromatography–mass spectrometry, and polar and semi-polar metabolites of bulbs were profiled by liquid chromatography–mass spectrometry. Membrane-stabilizing activity was evaluated using a hypotonic hemolysis assay. Potassium was the main macroelement, reaching 14,119 mg/kg dry weight in bulbs and 13,176 mg/kg in aerial parts. Magnesium and iron reached 4394 and 2435 mg/kg, respectively. Lead concentrations in bulbs of high-altitude species ranged from 0.75 to 4.47 mg/kg. Gas chromatography–mass spectrometry showed differences in n-alkane distributions, with some samples dominated by C_22_ and others extending to C_30_, reflecting species, developmental, and environmental variation. LC–MS/MS profiling revealed tentative assignments of diverse organosulfur compounds, amino acid derivatives, flavonoids, and oxygenated fatty-acid derivatives. Among the studied chloroform extracts, *A. suworowii* showed the highest membrane-stabilizing activity at a 10% concentration (51.20%). The four *Allium* taxa showed distinct elemental and metabolite profiles, although these differences were not subjected to a common statistical comparison across taxa. These results provide a comparative chemical foundation for future chemotaxonomic studies and for assessing the nutritional and pharmacological potential of wild *Allium* species.

## 1. Introduction

Central Asia is one of the major centers of *Allium* diversity, harboring approximately one-third of the world’s species diversity. In particular, the Tian Shan Mountains (in Kazakhstan), characterized by high endemism, include 56 endemic *Allium* species among the 16 major genera recorded in this mountain system [1]. In Kazakhstan’s flora, 9–21 endemic and 14 subendemic *Allium* species are listed, reflecting the considerable floristic value of this genus within the country [2,3].

Representatives of the genus *Allium* are recognized for their ornamental value, distinctive chemical profiles, essential nutrients, antioxidant potential and contribution to the prevention of oxidative stress-related disorders, including cardiovascular and chronic diseases [4,5,6,7]. However, despite the wide distribution of *Allium* species in Kazakhstan, their phytochemical characteristics and biological potential remain insufficiently investigated.

Medicinal properties have been reported for at least 12 *Allium* species occurring in Kazakhstan, including species distributed in the western part of the Kyrgyz Alatau (*A. karataviense* Regel, *A. obliquum* L., *A. sativum* L. (syn. *A. longicuspis* Regel), and *A. suworowii* Regel) [8]. In addition, at least fourteen *Allium* species in Kazakhstan are considered edible [9]. Previous studies have determined the taxonomic composition, conservation status, introduction potential, utilization, and prospects for further research of 134 *Allium* species in the flora of Kazakhstan, including 12 rare species. Nearly half of these species, representing ornamental, edible, medicinal, and melliferous plants, have been examined under cultivation conditions, demonstrating their potential for integrated scientific and practical investigations [10].

Phytochemical investigations in the literature have shown that sulfur-containing compounds are the primary classes of characteristic metabolites in *Allium* species, including alliin, allicin, ajoenes, vinyldithiins, and sulfides. Extracts and isolated compounds from *Allium* species have demonstrated various biological activities [11,12,13,14,15,16]. Nevertheless, the phytochemical composition and biological properties have been investigated for only a limited number of *Allium* species, representing no more than approximately 15–20% of the genus diversity [10]. Some species, including *Allium galanthum* and *A. turkestanicum*, have shown low toxicity and promising antimicrobial, antioxidant, and tyrosinase-inhibitory activities, indicating their potential as valuable plant raw materials [17,18].

Beyond secondary metabolites, the elemental composition of *Allium* species represents another important aspect of their biological and nutritional value. Species of the genus *Allium* have been reported to accumulate nutritionally important macro- and microelements, including Se, Fe, Zn, Mn, and K. For example, *A. angulosum* and *A. schoenoprasum* have been described as effective Se accumulators, with selenium concentrations depending on plant developmental stage and environmental conditions [19,20]. Mineral accumulation in plants is influenced by multiple ecological factors, including soil characteristics, altitude, climatic conditions, and species-specific physiological adaptations.

Despite the recognized chemical and biological diversity of *Allium* species in Kazakhstan, comparative studies integrating elemental composition, GC–MS-based profiling of lipophilic constituents, n-alkane chain-length distribution, and biological activity remain limited. Previous studies have generally focused on individual species or specific groups of compounds, whereas an integrated comparative assessment of these complementary chemical and biological characteristics among wild *Allium* species from Kazakhstan is still lacking.

This study aimed to comparatively characterize four wild *Allium* species from different subgenera (*Allium atrosanguineum* var. *fedschenkoanum* (Regel) G.H. Zhu &Turland, *A. trachyscordum* Vved., and *A. turkestanicum* Regel) and a rare species (*A. suworowii* Regel) from the Kazakh part of the Kyrgyz Alatau (Appendix A, Table A1). The objective of this study was to characterize the macro- and microelement composition of bulbs and to investigate the chemical composition of underground organs of selected *Allium* species from the Kyrgyz Alatau using elemental, GC–MS and LC-MS analyses.

## 2. Results

### 2.1. Elemental Profiles of Investigated Wild Allium Taxa

We measured concentrations of four macroelements (K, Ca, Mg, Na), five microelements (Fe, Mn, Zn, Cu, Ni) and two toxic elements (Pb, Cd), as well as ash content (%), in the aerial parts, bulbs, and peels of *Allium* species collected at various phenological stages (vegetative growth, flowering, fruiting, and senescence) (Table 1).

Potassium (K), calcium (Ca), and magnesium (Mg) were the predominant elements, whereas iron (Fe), manganese (Mn), zinc (Zn), copper (Cu), and nickel (Ni) were present at considerably lower concentrations. Elemental distribution showed marked organ-specific variation, with the direction and magnitude of differences varying among species. Bulbs generally contained higher concentrations of several major elements than aerial tissues, particularly in *A. atrosanguineum* var. *fedschenkoanum* and *A. turkestanicum*. However, this trend was not consistent across all species and elements. Overall, the observed elemental patterns show substantial variation by species and organ, but the current sampling design does not allow the independent effects of species, organ, altitude, and phenological stage to be separated.

Pronounced organ-specific differences were observed for calcium (Ca) and sodium (Na). In *A. turkestanicum*, the peel exhibited particularly high concentrations of Ca and K, whereas in *A. suworowii*, Ca was more abundant in aerial tissues than in the bulb. *A. trachyscordum* displayed a distinct Na distribution, with substantially higher Na concentrations in the aerial part than in the bulb. These findings demonstrate pronounced differences in mineral distribution among organs and species.

Trace-element profiles also varied considerably among the taxa. *A. atrosanguineum* var. *fedschenkoanum* exhibited comparatively high concentrations of Fe and Mn, particularly in the bulb, whereas these elements were generally less abundant in *A. suworowii* and *A. trachyscordum*. Zn, Cu, and Ni displayed lower overall concentrations and considerable organ-specific variation.

Lead (Pb) and cadmium (Cd) were detected in most analyzed samples, with substantial differences among species and organs. Pb concentrations (0.27–4.47 mg/kg DW) exceeded the pharmacopeial limit of 0.5 mg/kg in six samples. In contrast, Cd concentrations (0.02–0.49 mg/kg DW) were below the permissible limit of 0.5 mg/kg [21].

Ash content varied considerably among plant organs and phenological stages. The highest values were found in the bulb peel (18.32%) and bulbs (15.55%) of *A. turkestanicum*, while the lowest (2.93%) was observed in the aerial parts of flowering *A. trachyscordum*. In *A. atrosanguineum* var. *fedschenkoanum*, ash content reached 6.38% at the end of the growing season.

The elemental profiles demonstrate pronounced interspecific and organ-specific variation, with several notable deviations from the general pattern of bulb-dominant accumulation.

### 2.2. GC-MS Profiling of Chloroform Extracts from Four Studied Allium Bulbs

**Figure 1:**
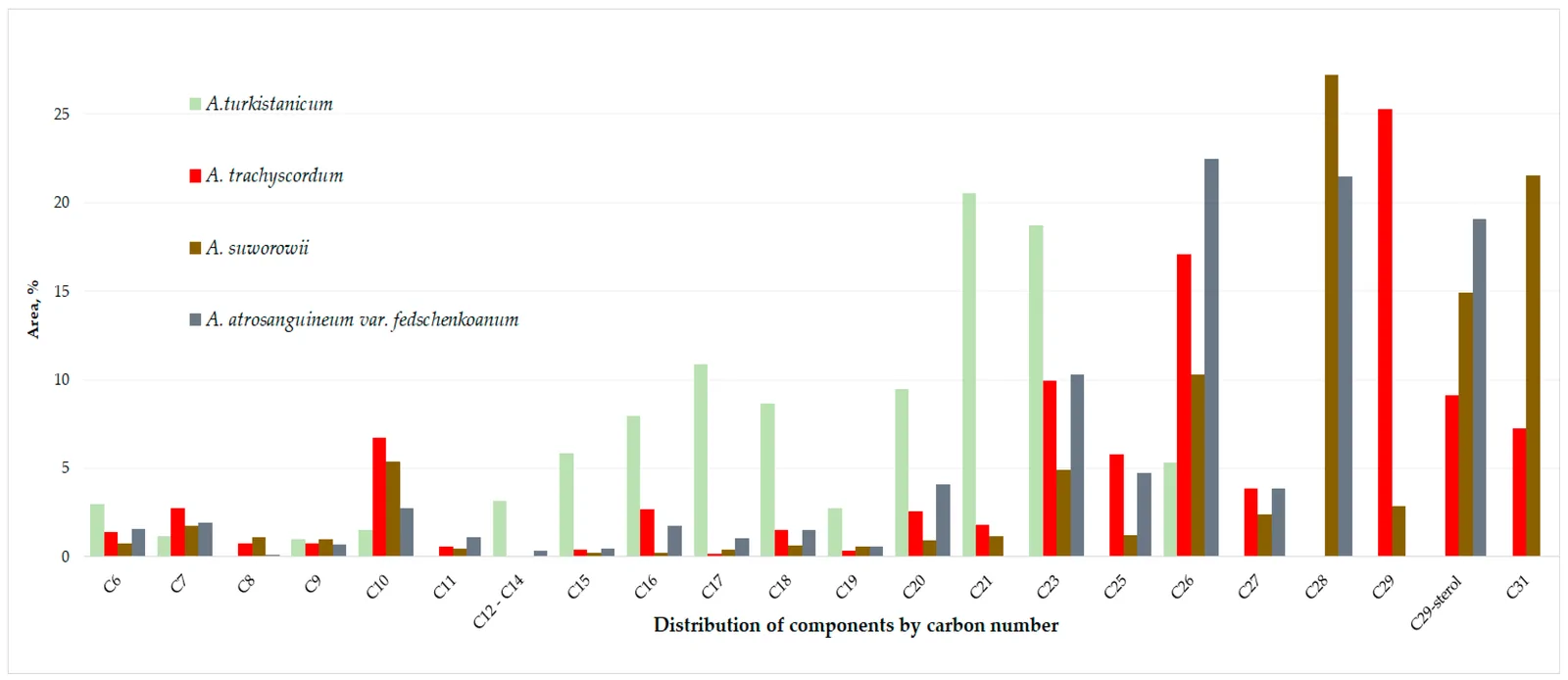
Distribution of molecular components by carbon number across altitude gradients and phenological stages.

GC-MS profiles were evaluated using the sum of peak areas. Relative abundances were calculated as each compound’s percentage of the total peak area. Compounds were identified by retention time (RT) and mass spectral library matching. Most detected compounds were n-alkanes, which were analyzed using their carbon chain length distribution (Table 2 and Table S1–S4, Figure 1).

Detected chemical constituents were grouped by carbon chain length to characterize variation in hydrocarbon profiles across studied species collected at different altitudes and phenological stages. Three main distribution patterns were determined.

*Allium turkestanicum* was characterized by a predominance of medium-chain homologues in the C_15_–C_23_ range (dodecanoic acid, undecyl ester (9.94%), heptadecane (9.18%), tricosane (8.79%), hexadecane (7.95%), 2-pentadecanone, 6,10,14-trimethyl- (6.46%), and heneicosane (6.29%)), with the highest contribution of docosane (20.57%). In contrast *A. atrosanguineum* var. *fedschenkoanum* showed an increased proportion of long-chain hydrocarbon homologues, with C_25_–C_29_ compounds collectively accounting for more than 60% of the detected hydrocarbon fraction. The most abundant individual compounds were pentacosane (22.48%), heptacosane (21.50%), and nonacosane (17.91%), while tricosane accounted for 10.27%.

The observed shift toward longer carbon chains may reflect species-specific and/or environmental and developmental differences. However, the present sampling design does not allow these effects to be separated.

Across the analyzed samples, the dominant carbon number gradually increased from shorter-chain homologues (C_22_) in the low-altitude early-stage sample to longer-chain compounds, including C_31_, in later developmental stages and higher-altitude habitats. This progressive shift suggests modification of n-alkane profiles, potentially reflecting adaptation of plant cuticular wax composition to changing environmental conditions and developmental requirements.

However, the analyzed taxa included four *Allium* species belonging to the different subgroups and were collected at different phenological stages along an altitudinal gradient; the observed differences should be interpreted cautiously. Further research is required to disentangle the relative contributions of species identity, developmental stage, and environmental factors.

### 2.3. LC–MS Profiling of the Investigated Allium Species

Four *Allium* bulb samples (*A. atrosanguineum* var. *fedschenkoanum, A. trachyscordum*, *A. turkestanicum* and *A. suworowii*) underwent LC–MS/MS analysis after cold extraction with acetonitrile/water (1:1, *v*/*v*). Data collection in both positive- and negative-ionization modes ensured comprehensive metabolite profiles. Each plant sample was analyzed as a single LC–MS/MS injection, and the results are presented as qualitative profiles rather than statistically replicated quantitative measurements. The resulting chromatographic profiles revealed substantial differences in signal distribution and intensity, highlighting pronounced chemical heterogeneity among the samples.

The early chromatographic region (~0.5–0.7 min) showed a high proportion of polar signals across all samples (Table S6). In *A. suworowii, A. atrosanguineum* var. *fedschenkoanum,* and *A. trachyscordum*, these signals accounted for approximately 50–67% of the profile, depending on ionization mode. Later-eluting regions displayed more distinct sample-specific differences. γ-Glutamyl-related signals at 5.4 min were most prominent in *A. atrosanguineum* var. *fedschenkoanum*, while steroidal saponin-related signals at 6.0 min and flavonoid-related signals at 6.6–6.7 min were most pronounced in *A. trachyscordum*, especially in positive-ionization mode.

*Allium trachyscordum* was distinguished by characteristic phenolic signals, including methylated flavonol glycoside and acylated hydroxycinnamic acid compounds. A prominent ion at *m*/*z* 684.3, detected at 5.95 and 6.07 min in negative-ionization mode, was tentatively identified as the formate adduct [M + HCOO]^−^ of isorhamnetin-3,4′-di-O-glucoside. Product ions at *m*/*z* 610.2, 577.1, 487.1, and 473.2 supported fragmentation involving glycosyl moieties through sequential neutral losses and cross-ring cleavages. The 14 Da difference between *m*/*z* 487.1 and 473.2 indicated a methylation-related structural variation.

Additional ions at *m*/*z* 427.2 and 413.2 were tentatively assigned to acylated hydroxycinnamic acid derivatives. The *m*/*z* 427.2 ion matched a caffeic acid malonyl hexoside. Product ions at *m*/*z* 362.0, 347.2, 340.2, 322.1, and 361 were consistent with fragmentation of sugar-containing structures via dehydration and cross-ring cleavage.

Structural assignments are tentative, as they rely on precursor mass and MS/MS fragmentation data without authentic reference standards.

#### LC-MS Profile *Allium suworowii*

LC–MS analysis in both positive- and negative-ionization modes revealed a chemically diverse metabolite profile dominated by sulfur-containing compounds, lipid-derived metabolites, and glycolipids. Most metabolites were detected within the first 7 min of chromatographic separation, reflecting the predominance of relatively polar constituents extracted by the aqueous–acetonitrile procedure (Table 3 and Table S5).

In negative-ionization mode, the chromatograms were characterized by abundant low-molecular-weight ions detected between RT 0.17 and 1.23 min. The most intense signals included *m*/*z* 112, 164, 186, 203, 218, 233, and 240, consistent with the presence of highly polar sulfur-containing metabolites. These ions were among the dominant peaks in the chromatograms and accounted for most early-eluting compounds.

LC-MS/MS profiling of *Allium suworowii* (Suvorov’s onion) was performed in both negative (ESI^−^)- and positive (ESI^+^)-ionization modes to establish a broad metabolomic fingerprint of its polar to mid-polar constituents. In the early-eluting fraction (Rt 0.467 min), an intense base peak at *m*/*z* 112.0 was detected in negative-ionization mode and numerous co-eluting organic metabolites (e.g., *m*/*z* 169.4, 186.1). Based on *m*/*z* mass matching, this ion was tentatively assigned as a sulfur-containing oxyanion. The absence of a corresponding signal in ESI^+^ mode is consistent with the preferential detection of inorganic sulfur anions under negative electrospray conditions; however, confirmation by authentic standard analysis would be required for identification.

Native and organosulfur precursors (CSOs) were identified via their corresponding protonated and deprotonated ions. These included methiin (S-methyl cysteine sulfoxide) and probably ethiin (S-ethyl cysteine sulfoxide, identified as a sodium adduct at *m*/*z* 186.1 [M + Na − 2H]^−^, which formed from the ion at *m*/*z* 249.0 [M − SO_2_] and produced a characteristic fragment at *m*/*z* 186.1, supporting its tentative assignment as a sulfur-containing amino acid derivative.

The presence of characteristic *Allium* flavonoid-related signals was detected in ESI(+), tentatively assigned to rutin (quercetin-3-O-rutinoside) based on agreement with the protonated molecular ion 611.2 [M + H]^+^, together with a radical molecular ion at *m*/*z* 610.4. Additional flavonoid-related ions were detected across both ionization modes, including signals at *m*/*z* 684.3, 577.0, 489.3, 425.1, 373.0, 355.0, 299.1, 256.3, 248.9 (A/B ring) and 139.0 (C/A ring), suggesting the presence of structurally diverse flavonoid-related derivatives.

A lipid marker, DGMG (18:2) (glycolipid-related lipid signals), was detected at Rt 5.667 min, exhibiting strict dual-polarity validation. It appeared as the protonated molecule [M + H]^+^ at *m*/*z* 679.7 and as its sodium adduct [M + Na]^+^ at *m*/*z* 701.6.

*Allium suworowii* displayed the presence of steroidal saponin-related compounds (Rt 5.9–6.2 min). In negative-ion mode, high-mass parent ions corresponding to intact steroidal glycosides were observed *m*/*z* 713.7. In positive-ion mode, these compounds underwent in-source deglycosylation, yielding characteristic steroidal sapogenin aglycones, specifically the base peak at *m*/*z* 413.2 [M + H]^+^ and its corresponding sodium adduct at *m*/*z* 435.3 [M + Na]^+^. These signals are consistent with steroidal sapogenin- or saponin-related structures. Therefore, assignment of a specific spirostanol or furostanol backbone remains tentative in the absence of authentic standards and additional structural confirmation.

### 2.4. Membrane-Stabilizing Activity of Allium Species Chloroform Extracts

The membrane-stabilizing activity of chloroform extracts obtained from the bulbs of four *Allium* species is presented in Table 4 and Table A2. All tested extracts exhibited protection against hypotonicity-induced erythrocyte hemolysis, with protection index (PI) values ranging from 17.74% to 51.20%.

The highest membrane-stabilizing activity among the tested extracts was observed for the chloroform extract of *A. suworowii* at a concentration of 10%, with a PI value of 51.20 ± 4.14%. At the studied concentration of 1%, the PI value was 41.62 ± 1.89% and the value at 10% was significantly higher than that at 1% (*p* < 0.05), indicating that the higher extract concentration was associated with increased membrane protection under the experimental conditions. The chloroform extract of *A. trachyscordum* demonstrated PI at 35.68 ± 2.04% and 30.68 ± 5.08%. The PI was numerically lower at 10% than at 1%; however, the difference was not statistically significant. Thus, increasing the concentration did not significantly enhance its membrane-protective effect. The extract of *A. atrosanguineum* var. *fedschenkoanum* showed 32.52 ± 2.06% at 1% and 24.28 ± 1.02% at 10%. The PI at 10% was significantly lower than that at 1% (*p* < 0.01), indicating that increasing the concentration reduced the membrane-stabilizing effect under the experimental conditions. The lowest membrane-stabilizing activity was observed for the chloroform extract of *A. turkestanicum*, with PI values of 19.01 and 17.74% at concentrations of 1% and 10%, respectively. Therefore, increasing the concentration did not significantly improve the protective effect.

Overall, the chloroform extract of *A. suworowii* demonstrated the highest membrane-stabilizing potential among the investigated extracts. Increasing its concentration from 1% to 10% significantly enhanced membrane protection. In contrast, *A. atrosanguineum* var. *fedschenkoanum* exhibited a significant reduction in PI at 10%, whereas *A. turkestanicum* and *A. trachyscordum* showed non-significant numerical decreases. These findings indicate that the effect of concentration on membrane protection was species-dependent.

## 3. Discussion

### 3.1. Elemental Composition

Mineral accumulation in *Allium* L. species is determined by species identity, plant organ, and developmental stage. Our results align with previous findings that potassium is the predominant macronutrient in *Allium* taxa, though its concentration varies among taxa [19,22]. In earlier studies that reported the highest potassium levels in leaves, we found that bulbs and peel can also serve as significant potassium storage organs. For instance, bulbs of *A. atrosanguineum* var. *fedschenkoanum* and *A. trachyscordum* contained higher potassium concentrations than their aerial parts, indicating species-specific nutrient allocation.

Calcium showed the greatest variability among the analyzed macronutrients. Concentrations in this study were much higher than those typically reported [23,24,25], with the highest values in the peel of *A. suworowii*. Bulbs of *A. atrosanguineum* var. *fedschenkoanum* also accumulated about 6.5 times more calcium than aerial tissues during senescence. These results suggest that underground storage organs and protective tissues are major calcium reservoirs, reflecting developmental remobilization and species-specific nutrient partitioning.

Magnesium concentrations were generally higher than those reported for *A. odorum* and *A. schoenoprasum*. While previous studies typically report magnesium levels in edible leaves [23], this study examined magnesium distribution in both the aerial parts and bulbs of *A. atrosanguineum* var. *fedschenkoanum* and other species.

Distinct micronutrient accumulation patterns were observed. Iron concentrations far exceeded those in most *Allium* species [25,26,27], with the highest levels in bulbs of *A. atrosanguineum* var. *fedschenkoanum*.

The distribution of trace elements (Zn, Cu, Ni, Pb, and Cd) did not exhibit a consistent organ-specific pattern. Comparable variability has been observed in wild *Allium* species, where trace element distribution is strongly influenced by genotype, soil geochemistry, and environmental conditions, rather than by a uniform pattern of organ allocation.

Among the potentially toxic elements, Pb exhibited pronounced species- and organ-dependent variation. In the present study, Pb concentrations ranged from 0.27 to 4.47 mg/kg, with relatively high concentrations detected in the bulbs of *A. atrosanguineum* var. *fedschenkoanum*, *A. trachyscordum*, and *A. turkestanicum*. This organ-specific distribution is consistent with previous studies showing that heavy-metal accumulation in *Allium* depends on both metal availability and plant organ. In hydroponically grown *A. cepa*, the authors of [19] reported that Pb and other metals accumulated progressively with increasing metal concentrations in the growth solution and showed marked differences among roots, bulbs, and leaves, with roots generally representing the main site of accumulation. Liu et al. [28] found that Pb in *A. sativum* accumulated predominantly in roots, with limited translocation to bulbs and shoots, indicating restricted Pb mobility within the plant.

The present findings therefore do not demonstrate a generalized Pb-bioaccumulation capacity in wild *Allium*, but rather indicate species- and organ-specific Pb retention. This interpretation is consistent with the findings of [29], who reported that onion cultivars grown in soils with elevated bioavailable Pb and Cd were not classified as bioaccumulators, although Pb concentrations exceeded regulatory limits in some cultivars. Thus, elevated Pb concentrations in individual *Allium* samples should be interpreted in the context of metal availability, soil properties, plant-specific uptake and retention mechanisms, and organ distribution, rather than as evidence of inherent Pb hyperaccumulation. The particularly high Pb concentration in the bulb peel of *A. turkestanicum* may reflect preferential retention in outer bulb tissues, potentially through binding to cell walls and other structural components. However, because soil metal concentrations, chemical speciation, and bioavailability were not assessed in the present study, the mechanisms underlying the observed Pb distribution cannot be established conclusively.

### 3.2. GC–MS and LC–MS Findings

GC–MS analysis revealed clear differences in the chloroform profiles of the studied *Allium* species. The observed differences in alkane diversities may be associated with differences in phenological stage and habitat conditions. The investigated samples originated from different altitudes (454–2666 m a.s.l.), where plants experience differences in temperatures, solar radiation, humidity, and water availability. Changes in the proportion of long-chain alkanes in epicuticular may reflect environmental adaptation, particularly through modification of cuticle properties affecting water retention and protection against stress [30,31]. The major C_22_–C_29_ alkanes, including pentacosane, heptacosane, octacosane, and nonacosane, are commonly reported constituents of plant wax fractions [32,33].

The distribution of alkanes varied considerably among samples, suggesting differences in wax biosynthesis associated with species identity, developmental stage, and ecological conditions. *A. turkestanicum* was characterized by a higher proportion of shorter-chain hydrocarbons, particularly docosane, whereas samples from higher elevations showed greater abundance of very-long-chain alkanes (C_25_–C_29_). *Allium suworowii* and *A. trachyscordum* showed high levels of heptacosane and octacosane, respectively, while sample (2666) exhibited a balanced C_25_–C_29_ profile. Aldehydes and fatty-acid derivatives represented minor contributions. Among the analyzed samples, *A. trachyscordum* exhibited the greatest aldehyde diversity, including 2,4-decadienal and 2-decenal, which may reflect enhanced lipid turnover and oxidation of unsaturated fatty acids [34].

LC–MS/MS analysis revealed distinct chemical profiles for each sample. *Allium atrosanguineum* var. *fedschenkoanum* had more γ-glutamyl-related signals, *A. suworowii* showed a high proportion of early-eluting polar signals, *A. trachyscordum* featured pronounced flavonoid- and saponin-type signals, and *A. turkestanicum* displayed characteristic glycosylated flavonoid and acylated phenolic features.

Metabolic profiling of *A. suworowii* using dual-polarity LC–MS provided a comprehensive overview of its polar and semi-polar metabolites, identifying characteristic sulfur-containing compounds alongside various secondary metabolite classes (including S-alk(en)yl cysteine sulfoxide (CSO)-related compounds such as methiin-, ethiin-, and alliin-related ions, as well as γ-glutamyl peptide derivatives) which align with [35,36].

Sulfur-containing metabolites in *Allium* species form a chemically diverse network, ranging from sulfur intermediates to cysteine sulfoxides and organosulfur derivatives [37].

Additionally, the LC–MS profile also revealed flavonoid glycosides, glycolipid-related compounds, and steroidal saponin derivatives. The detection of rutin-like flavonoid ions supports previous findings that flavonoid glycosides are common in *Allium* species and contribute to their antioxidant and protective properties. Lipid-associated ions consistent with DGMG-type compounds indicate the presence of membrane-related metabolites. Steroidal saponin-related ions, characterized by presence of spirostanol or furostanol-type derivatives, are typical secondary metabolites in this genus [36].

### 3.3. Membrane Stabilizing Activity

Chloroform-soluble fractions from four *Allium* L. species (*A. turkestanicum*, *A. trachyscordum*, *A. atrosanguineum* var. *fedschenkoanum*, and *A. suworowii*) showed distinct differences in membrane-stabilizing activity. The observed differences in membrane-protective activity should be interpreted in the context of the limitations of the GC–MS-based chemical characterization. The compounds discussed here were tentatively assigned based on library matching with a threshold of ≥80% and therefore represent tentative rather than confirmed identifications. Compounds with lower similarity scores were not included in the reported compositional profile because their annotation was considered insufficiently reliable. However, their exclusion from the reported profile does not imply that they were absent from the extracts or biologically inactive. Consequently, the membrane-protective effects observed in the present study may reflect the combined contribution of both tentatively identified compounds and additional constituents that could not be reliably annotated using the applied GC–MS approach.

*Allium suworowii* showed relatively high membrane-protective effect, with the highest PI value (51.20%) at a 10% concentration, with a significant difference between the tested concentrations. Indomethacin, used as a reference, showed a PI of 65.13%. Notably, only *A. suworowii* demonstrated an increased membrane-protective effect with increasing extract concentration. Similarly, the decrease in protective activity at higher concentrations observed for samples of *A. turkestanicum*, *A. trachyscordum*, and *A. atrosanguineum* var. *fedschenkoanum* may reflect concentration-dependent changes in the combined effects of multiple extract constituents. The presence of relatively abundant aldehydes and other hydrophobic compounds provides a possible chemical context for these differences, but the current data cannot establish their individual contributions.

Therefore, the GC–MS results should be considered a preliminary chemical framework for interpreting the biological assay rather than evidence of specific compound–activity relationships. Further fractionation, compound-level identification using authentic standards, and testing of individual fractions or purified compounds would be needed to determine which constituents contribute to the observed membrane-protective effects.

## 4. Materials and Methods

### 4.1. Plant Material Preparation and Element Analysis

The studied plants were collected during expedition trips in April–August 2024–2025 at the different vegetation stages in the Zhambyl region, in three different districts, T. Ryskulov, Zhambyl and Talass, at different altitudes (Table 5 and Table A1). Four *Allium* L. [38] species were collected, with each plant serving as an individual biological sample. Samples were not pooled and were processed independently. Voucher specimens representing the collected taxa were deposited in the AA herbarium under the following accession numbers: AA0114456, AA0114458, AA0114457, and AA0003685. Species identification was based on diagnostic morphological features using the keys described in [39,40]. The scientific names of species are presented according to Plants of the World Online [41].

The assessed species were sampled during field trips at the phenological stage, which naturally occurred at the time of collection. Repeated sampling of each species throughout the growing season was beyond the study’s logistical scope; the influence of developmental stage cannot be separated from species-specific variation.

Plant samples were dried to constant weight, ground into a homogeneous powder, and used for mineral element analysis. Approximately 3–5 g of dried material was placed in porcelain crucibles and incinerated in a muffle furnace at 500–550 °C until a constant mass was reached. The ash was then cooled in a desiccator and weighed to determine total ash content [21].

For elemental analysis, ash samples were dissolved in concentrated nitric acid (HNO_3_) 2–5 mL and diluted up to 25 mL with ultrapure water. The solutions were filtered before instrumental analysis. Element concentrations were determined using atomic absorption spectrometry (Shimadzu AA-6200) [42,43]. Concentrations were calculated with external calibration curves from certified multi-element standard solutions and expressed as mg kg^−1^ dry weight. All analyses were performed in triplicate, and the results are presented as mean values ± standard deviation.

### 4.2. GC-MS Analysis

The chemical composition of chloroform extracts was analyzed using gas chromatography coupled with mass spectrometry (GC-MS). The analyses were carried out on an Agilent 7890A gas chromatograph equipped with a 5975C mass-selective detector and a DB-17ms capillary column (30 m × 0.25 mm, film thickness 0.25 µm). Helium (99.995% purity) was used as the carrier gas at a constant flow rate of 1.0 mL/min. Samples were injected in split mode (25:1) at a 1.0 µL injection volume. The injector temperature was maintained at 250 °C. The oven temperature program was set as follows: initial temperature of 40 °C (held for 3 min), increased at 5 °C/min to 250 °C, and held at 250 °C for 3 min. The transfer line, ion source, and quadrupole temperatures were 26 °C, 23 °C, and 15 °C, respectively. Electron impact ionization (EI) at 70 eV was applied, and mass spectra were recorded in the *m*/*z* range of 40–550.

Compounds were tentatively identified by comparing the mass spectra obtained with the NIST17 library. Only those with a library match of 80% or higher were included. Retention indices were not calculated because n-alkane standards were not used. Compound abundances were estimated from relative peak areas. Chromatographic data were processed with Agilent MassHunter Unknowns Analysis (version 10.1), and all peak assignments were checked manually.

### 4.3. LC–MS/MS Analysis

To isolate polar and semi-polar sulfur-containing compounds, plant material was defatted by cold hexane extraction (20 mL, 10 min, 2 extraction cycles), then extracted with acetonitrile–water (1:1, *v*/*v*). Extracts were filtered through 0.22 μm regenerated cellulose syringe filters before analysis. The LC–MS method was optimized through repeated analyses of prepared extracts before analysis of the study samples. For the final LC–MS analysis, 2 g of powdered plant material was extracted with 15 mL of distilled water under continuous stirring for 15 min, followed by the addition of 15 mL of HPLC-grade acetonitrile. The samples were centrifuged, and the supernatants were filtered through 0.22 μm RC syringe filters. The extracts were diluted ten-fold with HPLC-grade acetonitrile before injection [44,45].

LC–MS/MS analysis was performed using an Agilent 1200 liquid chromatography system (Agilent Technologies, Santa Clara, CA, USA) coupled to a SCIEX Triple Quad™ 3500+ mass spectrometer (SCIEX, (AB Sciex Pte. Ltd., Singapore, Singapore)equipped with an electrospray ionization (ESI) source. Measurements were performed in both positive- and negative-ionization modes.

Chromatographic separation was carried out using a Phenomenex Synergi™ Fusion-RP C18 column (50 × 2.0 mm, 4 μm, 80 Å (Phenomenex, Torrance, CA, USA) maintained at 30 °C. The mobile phase consisted of water (solvent A) and acetonitrile (solvent B), delivered at a flow rate of 0.2 mL min^−1^. The injection volume was 20 μL. The optimized gradient program was: 5–20% B (0–3 min), 20–30% B (3–5 min), 30–50% B (5–8 min), 50–100% B (8–10 min), 100% B (10–12 min), followed by re-equilibration to 5% B (12–15 min).

Mass spectra were acquired in both positive- and negative-ESI modes. Nitrogen was used as the nebulizing, curtain, heater, and collision gas. The optimized source parameters were: capillary temperature, 250 °C; spray voltage, 4.50 kV; and ion spray potentials of +3 and −47 V for positive- and negative-ionization modes, respectively. Data were collected within an *m*/*z* range of 80–900.

Raw LC–MS data were processed using SCIEX Analyst^®^ software (version 1.6). Chromatographic peaks were evaluated based on their precursor/product ion transitions and MS/MS fragmentation patterns, where available. Compounds were tentatively assigned by comparison with published mass spectrometric and fragmentation data, as well as with available reference data. The LC–MS/MS data were used for semi-qualitative profiling and comparison of relative signal intensities; no absolute quantification or confirmation with authentic standards was performed.

### 4.4. Membrane-Stabilizing Activity Assay

Membrane-stabilizing activity was assessed using a hypotonicity-induced erythrocyte hemolysis assay. Peripheral blood was voluntarily donated by one author, who served as a healthy donor and provided written informed consent for the collection and use of the blood sample in this study. Erythrocytes were washed three times with phosphate-buffered saline (PBS, pH 7.4) by centrifugation at 2500 rpm for 15 min and then prepared as a 2% erythrocyte suspension.

Extract solutions at concentrations of 1% and 10% were used. For each assay, 1 mL of the extract solution was mixed with 20 μL of the erythrocyte suspension and 4 mL of either PBS to prepare the isotonic sample or distilled water to prepare the hypotonic sample. The samples were incubated at 37 ± 1 °C for 60 min and subsequently centrifuged at 2500 rpm for 15 min. Hemolysis was quantified by measuring the absorbance of the supernatant at 540 nm [46].

The protection index (PI, %) was calculated using the following equation:

PI (%) = [1 − (OD2 − OD1)/(OD3 − OD1)] × 100,

where OD1 represents the absorbance of the isotonic sample containing PBS, extract, and erythrocytes; OD2 represents the absorbance of the hypotonic sample containing distilled water, extract, and erythrocytes; and OD3 represents the absorbance of the complete-hemolysis control containing distilled water and erythrocytes, corresponding to 100% hemolysis.

Indomethacin was tested as the reference drug using 1% and 10% solutions under the same experimental conditions. The membrane-stabilizing activity of the extracts was determined relative to the complete-hemolysis control and was additionally compared with that of indomethacin.

All experiments were performed in triplicate, and the results were expressed as the mean ± standard deviation. Differences between the 1% and 10% solutions within each species and for indomethacin were analyzed using Welch’s unpaired *t*-test. A *p*-value of less than 0.05 was considered statistically significant.

## 5. Conclusions

This study compares the elemental composition and metabolite profiles of four wild *Allium* species from Kyrgyz Alatau, Kazakhstan. Elemental accumulation varied among species, plant organs, and phenological stages, with potassium and calcium as the main macroelements. While cadmium concentrations were within permissible limits, several samples showed elevated lead levels, underscoring the need for safety monitoring of wild edible and medicinal plants. GC–MS profiling revealed distinct differences in alkane distributions, while LC–MS/MS revealed substantial variation in bulb metabolites, including sulfur-containing and lipid-derived compounds. These results enhance the phytochemical characterization of Central Asian *Allium* species and support future chemotaxonomic and bioactivity research. Further studies with replicated sampling, targeted metabolomics, authentic standards, and quantitative analyses are recommended to confirm metabolite identities and expand their biological value.

## Figures and Tables

**Table 1:** Qualitative and quantitative analyses of wild *Allium* species from the Kyrgyz Alatau, Zhambyl region, Kazakhstan (mg/kg ± SD).

Elements	*A. atrosanguineum* var. *fedschenkoanum* (Senescence Phase)		*A. turkestanicum* Vegetation			*A. suworowii* Beginning of Fruiting			*A. trachyscordum* Flowering	
	Aerial	Bulb	Aerial	Bulb	Peel	Aerial	Bulb	Peel	Aerial	Bulb
K	5273.20 ± 35.3	8046.11 ± 28.9	13,175.68 ± 30.5	7487.53 ± 33.4	14,119.17 ± 23.2	8978.91 ± 28.9	10,343.32 ± 42.3	3484.11 ± 26.4	6461.28 ± 18.3	2768.28 ± 38.4
Ca	2586.04 ± 28.9	16,926.99 ± 25.7	16,497.46 ± 23.4	1115.99 ± 24.6	31,854.23 ± 19.7	3898.42 ± 35.2	597.11 ± 28.3	10,621.61 ± 29.7	2119.02 ± 28.4	2362.83 ± 32.1
Mg	930.60 ± 16.7	4394.45 ± 13.2	2796.81 ± 14.2	839.60 ± 18.4	2843.78 ± 14.3	1275.40 ± 11.8	683.27 ± 12.2	1167.98 ± 13.2	552.83 ± 12.8	368.18 ± 13.2
Na	418.25 ± 8.2	426.90 ± 7.8	1438.27 ± 8.4	579.98 ± 9.2	986.25 ± 10.2	1084.36 ± 13.4	1381.70 ± 8.9	862.58 ± 10.2	1675.93 ± 10.0	125.08 ± 11.3
Fe	1073.79 ± 5.6	2435.36 ± 8.4	358.82 ± 9.6	98.12 ± 7.4	1577.67 ± 7.8	106.36 ± 6.5	45.61 ± 7.3	110.88 ± 7.3	221.78 ± 9.6	137.91 ± 7.9
Mn	83.64 ± 0.07	223.56 ± 0.08	59.29 ± 0.08	19.13 ± 0.6	83.90 ± 0.01	8.70 ± 0.1	7.35 ± 0.08	14.51 ± 0.09	33.88 ± 1.0	18.05 ± 0.8
Zn	21.21 ± 0.09	39.84 ± 1.5	4.28 ± 1.8	6.56 ± 0.9	7.17 ± 1.3	12.74 ± 0.09	18.33 ± 0.19	18.33 ± 0.08	21.35 ± 1.0	17.88 ± 2.3
Cu	4.12 ± 0.15	7.66 ± 0.23	3.67 ± 0.08	1.88 ± 0.9	5.83 ± 1.2	1.25 ± 0.7	2.33 ± 0.08	0.83 ± 0.02	5.20 ± 0.0	3.85 ± 0.08
Ni	4.58 ± 0.06	5.14 ± 0.15	3.37 ± 0.08	1.56 ± 0.08	6.23 ± 0.02	1.46 ± 0.05	0.73 ± 0.01	1.87 ± 0.03	3.17 ± 0.08	0.51 ± 0.15
Pb	0.98 ± 0.04	2.71 ± 0.07	1.02 ± 0.08	0.31 ± 0.01	4.47 ± 0.08	0.40 ± 0.05	0.75 ± 0.05	0.27 ± 0.04	3.61 ± 0.08	-
Cd	0.47 ± 0.01	0.32 ± 0.05	0.39 ± 0.03	0.15 ± 0.04	0.49 ± 0.01	0.02 ± 0.0	-	0.16 ± 0.01	0.42 ± 0.01	0.2 ± 0.03
Ash. %	6.38 ± 2.8	15.00 ± 3.2	11.62 ± 2.6	15.55 ± 1.8	18.32 ± 1.6	4.85 ± 1.3	3.53 ± 1.2	5.39 ± 1.8	2.93 ± 1.1	4.03 ± 1.8

**Table 2:** Distribution of components by carbon number in the four studied *Allium* samples (bulbs) (relative peak areas, %).

Cn	RT, Min	*A. turkestanicum*	*A. trachyscordum*	*A. suworowii*	*A. atrosanguineum* var. *fedschenkoanum*
C_6_	5.7–7.6	2.96	1.40	0.77	1.58
C_7_	9.2–11.2	1.17	2.72	1.77	1.95
C_8_	5.5–27.3	—	0.74	1.08	0.13
C_9_	12.3–20.6	0.98	0.73	0.99	0.67
C_10_	19.1–22.3	1.49	6.74	5.39	2.73
C_11_	23.5	—	0.55	0.48	1.10
C_12_–C_14_	24.3–31.8	3.16	—	—	0.37
C_15_	26.8–31.8	5.85	0.43	0.20	0.46
C_16_	29.1–37.6	7.95	2.67	0.21	1.77
C_17_	31.4–36.1	10.87	0.17	0.40	1.05
C_18_	33.5–34.5	8.68	1.50	0.62	1.52
C_19_	35.5	2.75	0.32	0.58	0.58
C_20_–C_21_		9.48	2.59	0.93	4.10
		20.57	1.83	1.15	—
C_23_		18.73	9.96	4.94	10.27
C_24_		—	5.78	1.22	4.75
		5.34	17.08	10.32	22.48
C_26_		—	3.85	2.39	3.88
C_27_		—	—	27.24	21.50
C_28_		—	25.32	2.89	—
C_29_		—	9.13	14.91	19.11
C_31_		—	7.26	21.53	—

The data were calculated by summing the peak areas of all compounds with the same number of carbon atoms in their formulas.

**Table 3:** Tentative assignments of major chromatographic peaks detected in positive- and negative-ionization modes of *Allium suvorowii*.

Rt (Min)	Compounds	Class	Mode (s)	Observed *m*/*z*	*m*/*z* (Δ)	Major Fragment Ions (*m*/*z*)
0.584	S-propenyl-L-cysteine	Organosulfur	ESI(−)	196.4		155; 112
0.701	S-methylcysteine sulfoxide	Methiin	ESI(−) ESI(+)	150.1/152.0	(−0.08)/(+0.04)	ESI(−): 130.0; 112.0; ESI(+): 136.0, 120.0
	Phenylalanine	Amino acid	ESI(−)	164.1	(+0.2)	147.0; 103.0
	Tryptophan	Amino acid	ESI(−)	203	(+0.1)	159.0; 142.0; 116.0
5.66–5.95	S-containing acid derivative (S-(2-carboxypropyl) cysteine sulfoxide-related, tentative)	Organosulfur	ESI(−)	249.0		186.1; 223.2; 139.0
	DGMG (18:2)	Glycolipid	ESI(+)	679.7 [M + H]^+^		614.5; 453.3; 340.2
				701.6 M + Na]^+^		
	Steroidal saponin	Saponin	ESI(−)	713.7		551.6; 413.2 (ESI^+^)
6.19–6.25	HOME-type	Oxylipin	ESI(−)	297.0–297.2	(−0.1–0.2)	279.2; 171.1
≃6.37	Oxygenated fatty-acid derivative (diHODE-type oxylipin, tentative)	Oxylipin	ESI(−)	430.9/481.1	(<0.02)	311.2; 297.2; 248.9

**Table 4:** Membrane-stabilizing activity of chloroform extracts from *Allium* species.

Sample	Concent., %	PI (% ± SD)	Interpretation
*A. turkestanicum*	1	19.01 ± 1.07	The PI value was numerically lower than that observed at 1%; increasing the concentration did not enhance the protective effect.
	10	17.74 ± 3.05	
*A. trachyscordum*	1	35.68 ± 2.04	The PI value was numerically lower at 10% than at 1%.
	10	30.68 ± 5.08	
*A. atrosanguineum* var. *fedschenkoanum*	1	32.52 ± 2.06	
	10	24.28 ± 1.02 **	
*A. suworowii*	1	41.62 ± 1.89	The highest PI value among the tested extracts was observed at 10% rather than at 1%.
	10	51.20 ± 4.14 *	

Note: Values are expressed as the mean ± SD (*n* = 3). * *p* < 0.05 and ** *p* < 0.01 compared with the corresponding 1% concentration within the same species, according to Welch’s unpaired *t*-test.

**Table 5:** Representatives of *Allium* species studied.

Species	GPS Data	Altitude, m Above Sea Level	Date of Collection	Voucher
*Allium atrosanguineum* var. *fedschenkoanum* (Regel) G.H. Zhu & Turland	N 42°40′07.6″ E 72°46′48.9″	2666	22 August 2025	AA0114456
*A. suworowii* Regel	N 42.8651568 E 71.9170940	1182	3 May 2025	AA0114458
*A. trachyscordum* Vved.	N 42.88742504 E 71.82125881	1052	5 May 2025	AA0114457
*A. turkestanicum* Regel	N 43°25′29″ E 70°34′43″	454	23 April 2024	AA0003685

## Data Availability

The original contributions presented in this study are included in the article/Supplementary Materials. Further inquiries can be directed to the corresponding author.

## Supplementary Materials

The following supporting information can be downloaded at https://www.mdpi.com/article/10.3390/molecules31183266/s1, Table S1: GC-MS profile of chloroform extract of *Allium atrosanguineum* var. *fedschenkoanum* bulb (senescence phase) (H 2666 a.s.l.); Table S2: GC-MS profile of chloroform extract of *A. suworowii* Regel bulb (H 1182 a.s.l.); Table S3: GC-MS profile of chloroform extract of *A. trachyscordum* Vved. bulb (H 1052 a.s.l.); Table S4: GC-MS profile of chloroform extract of *A. turkestanicum* Regel bulb (H 454 a.s.l.); Table S5: Mass spectrometric characteristics of detected compounds in positive- and negative-electrospray-ionization modes (ESI^+^/ESI^−^) of *A. suworowii* Regel bulb; Table S6: Comparison of dominant peaks across studied *Allium* L. bulb samples.

## Appendix A

**Table A1:** Taxonomic classification, morphological characteristics, flowering period, and habitat preferences of the investigated *Allium* species.

Species	Subgenus/Section	Stem, cm	Bulbs	Flowering Period	Habitats
*A. atrosanguineum var. fedschenkoanum* (Regel) G.H. Zhu &Turland	Cepa (Mill.) Prokh./Annuloprason T.V. Egorova	15–50	Solitary or clustered in small groups on a short rhizome, oblong–ovoid, poorly developed, 0.5–1.0 cm wide. Outer tunics brown, coarsely fibrous.	June–August	Rocky slopes and fine-soil habitats in the alpine and subalpine mountain belts, often forming extensive populations.
*A. turkestanicum* Regel	Allium/Mediasia F. O. Khass., Yengal. & N. Friesen	50–100	Subglobose, 1.5–3.0 cm wide. Outer tunics membranous, papery, grayish, without distinct veins.	June–July	Clayey, sandy, and gravelly desert steppes.
*A. suworowii* Regel	Melanocrommyum (Webb & Berth.) Rouy/Acmopetala R.M. Fritsch	40–100	Globose, 2–3 cm in diameter, with gray, leathery, splitting outer tunics.	May–June	Shrub-covered and herbaceous mountain slopes and colluvial foothills.
*A. trachyscordum* Vved.	Reticulatobulbosa (Kamelin) N. Friesen/Scabriscapa (Tscholok.) N. Friesen	25–35	Clustered in small groups on a short ascending rhizome, cylindric–conical, with brown, reticulate outer tunics.	June–July	Gravelly mountain slopes.

**Table A2:** Absorbance values and membrane-stabilizing activity of chloroform extracts from *Allium* species determined by the hypotonicity-induced erythrocyte hemolysis assay.

Species	Concentration, %	OD_1_ (PBS + Extract + RBCs)	OD_2_ (H_2_O + Extract+ RBCs)	PI, %
*A. turkestanicum* Regel	1	0.044 ± 0.003	0.372 ± 0.001	19.01 ± 1.07
	10	0.060 ± 0.001	0.380 ± 0.002	17.74 ± 3.05
*A. trachyscordum* Vved.	1	0.051 ± 0.000	0.307 ± 0.001	35.68 ± 2.04
	10	0.097 ± 0.002	0.341 ± 0.001	30.68 ± 5.08
*A. atrosanguineum* var. *fedschenkoanum* (Regel) G.H. Zhu & Turland	1	0.040 ± 0.001	0.316 ± 0.001	32.52 ± 2.06
	10	0.033 ± 0.001	0.348 ± 0.003	24.28 ± 1.02 **
*A. suworowii* Regel	1	0.079 ± 0.001	0.295 ± 0.001	41.62 ± 1.89
	10	0.074 ± 0.001	0.257 ± 0.017	51.20 ± 4.14 *
Reference (indomethacin)	1	0.036 ± 0.001	0.232 ± 0.000	52.00 ± 4.13
	10	0.025 ± 0.001	0.165 ± 0.001	65.13 ± 3.22 *

Notes: OD_1_—absorbance of the isotonic sample containing PBS, extract, and RBCs; OD_2_—absorbance of the hypotonic sample containing H_2_O, extract, and RBCs; and OD_3_—complete-hemolysis control containing H_2_O and RBCs (OD_3_ = 0.449). PBS, phosphate-buffered saline; RBCs, red blood cells. The protection index was calculated using the following equation: PI (%) = [1 − (OD2 − OD1)/(OD3 − OD1)] × 100. Values are expressed as the mean ± SD (*n* = 3). * *p* < 0.05 and ** *p* < 0.01 compared with the corresponding 1% concentration within the same species or reference drug, according to Welch’s unpaired *t*-test.
