# Supplementary material for: Integrated Chemical and Biological Profiling of Selected Wild Allium Species from Kyrgyz Alatau
Source: Molecules. 2026 Sep 15;31(18):3266. doi: 10.3390/molecules31183266 (PMC13609529; doi:10.3390/molecules31183266)
Supplement: Supplementary file 1 [file molecules-31-03266-s001.zip › molecules-4523745-supplementary-final.pdf]

Table S1. GC-MS profile of chloroform extract of Allium *atrosanguineum* var. *fedschenkoanum* bulb (senescence phase) (H 2666 a.s.l.).

5,75	Hexanal	C6H12O	1,576318816
11,22	2-Heptenal, (E)-	C7H12O	1,952625157
14,67	2-Octenal, (E)-	C8H14O	0,12848254
15,89	4-Nonenal, (E)-	C9H16O	0,261141358
16,07	Nonanal	C9H18O	0,203320136
19,12	Decanal	C10H20O	0,342835668
20,61	4-Oxononanal	C9H16O2	0,213491884
20,76	2-Decenal, (E)-	C10H18O	0,562102422
21,05	Cyclohexanone, 2-butyl-	C10H18O	0,239858048
21,74	2,4-Decadienal, (E,E)-	C10H16O	0,619633541
22,37	2,4-Decadienal, (E,E)-	C10H16O	0,967292178
23,52	2-Undecenal	C11H20O	1,096774969
26,11	1-Hexanone, 1-phenyl-	C12H16O	0,126058968
26,81	Dodecane, 2,6,11-trimethyl-	C15H32	0,464141172
29,16	Hexadecane	C16H34	0,720374949
31,39	Heptadecane	C17H36	1,045544532
31,78	Cyclododecanemethanol	C13H26O	0,240262984
33,51	Octadecane	C18H38	0,443323235
34,5	2-Pentadecanone, 6,10,14-trimethyl-	C18H36O	1,081045929
35,53	Nonadecane	C19H40	0,576030405
37,57	n-Hexadecanoic acid	C16H32O2	1,052594648
39,31	Heneicosane	C21H44	2,493840139
41,95	Isopropyl linoleate	C21H38O2	1,611430386
42,79	Tricosane	C23H48	10,26728007
44,41	Tetracosane	C24H50	4,746250837
46,01	Pentacosane	C25H52	22,48203898
47,49	Hexacosane	C26H54	3,877856702
48,97	Heptacosane	C27H56	21,49933204
52,38	Nonacosane	C29H60	17,90701862
53,26	Stigmasterol	C29H48O	1,2016987

Table S2. GC-MS profile of chloroform extract of *A. suworowii* Regel bulb (H 1182 a.s.l.).

5,5	Octane	C8H18	0,425595
5,74	Hexanal	C6H12O	0,766628
11,23	2-Heptenal, (E)-	C7H12O	1,770675
14,07	3-Octen-2-one	C8H14O	0,28014
14,66	2-Octenal, (E)-	C8H14O	0,367235
16,07	Nonanal	C9H18O	0,558079
17,82	trans-2-Nonenal	C9H16O	0,429604
18,92	cis-4-Decenal	C10H18O	0,180396
20,77	2-Decenal, (E)-	C10H18O	3,195735
21,74	2,4-Decadienal, (E,E)-	C10H16O	0,749632
22,37	2,4-Decadienal, (E,E)-	C10H16O	1,25517
23,52	2-Undecenal	C11H20O	0,481081
29,16	Hexadecane	C16H34	0,206235
31,39	Heptadecane	C17H36	0,404338
31,78	Pentadecanal-	C15H30O	0,203281
33,51	Nonadecane	C19H40	0,25271
34,49	2-Pentadecanone, 6,10,14-trimethyl-	C18H36O	0,391542
35,53	Nonadecane	C19H40	0,326679
37,42	Hexadecanoic acid, ethyl ester	C18H36O2	0,229878
39,3	Heneicosane	C21H44	0,929907
42,78	Tricosane	C23H48	4,944515
44,42	Tetracosane	C24H50	1,218236
44,93	1,21-Docosadiene	C22H42	1,152165
45,98	Pentacosane	C25H52	10,32399
47,48	Hexacosane	C26H54	2,385686
47,74	Hentriacontane	C31H64	5,271431
47,92	Hentriacontane	C31H64	16,25904
48,99	Heptacosane	C27H56	27,24085
50,48	Octacosane	C28H58	2,888215
52,38	Nonacosane	C29H60	13,1523
53,24	Stigmasterol	C29H48O	1,759034

Table S3. GC-MS profile of chloroform extract of *A. trachyscordum* Vved. bulb (H 1052 a.s.l.).

5,52	Octane	C8H18	0,32739
5,74	Hexanal	C6H12O	1,081409
7,6	2-Hexenal, (E)-	C6H10O	0,323707
11,22	2-Heptenal, (E)-	C7H12O	2,718083
14,07	3-Octen-2-one	C8H14O	0,196049
14,66	2-Octenal, (E)-	C8H14O	0,321443
15,89	4-Nonenal, (E)-	C9H16O	0,11955
16,07	Nonanal	C9H18O	0,286788
18,92	cis-4-Decenal	C10H18O	0,27582
20,78	2-Decenal, (E)-	C10H18O	2,623471
21,75	2,4-Decadienal, (E,E)-	C10H16O	1,367344
22,38	2,4-Decadienal, (E,E)-	C10H16O	2,195048
23,52	2-Undecenal	C11H20O	0,546886
26,81	Pentadecane	C15H32	0,202119
27,27	Vanillin	C8H8O3	0,214732
29,16	Hexadecane	C16H34	0,133486
29,61	2-Propanone, 1-(4-hydroxy-3-methoxyphenyl)-	C10H12O3	0,273065
31,39	Heptadecane	C17H36	0,166871
31,78	Pentadecanal-	C15H30O	0,22985
33,51	Octadecane	C18H38	0,192469
34,51	2-Pentadecanone, 6,10,14-trimethyl-	C18H36O	0,79303
35,53	Nonadecane	C19H40	0,316861
37,22	Ethyl 9-hexadecenoate	C18H34O2	0,146757
37,43	Hexadecanoic acid, ethyl ester	C18H36O2	0,374435
37,63	n-Hexadecanoic acid	C16H32O2	2,537643
39,31	Heneicosane	C21H44	1,067488
41,09	Docosane	C22H46	1,261789
42,83	Tricosane	C23H48	9,27374
43,85	4,8,12,16-Tetramethylheptadecan-4-olide	C21H40O2	0,756555
44,45	Tetracosane	C24H50	3,225663
44,94	1,21-Docosadiene	C22H42	0,570409
45,83	.gamma.-Sitosterol	C29H50O	0,720586
46,05	Pentacosane	C25H52	16,31588
46,51	Docosanoic acid, methyl ester	C23H46O2	0,688109
47,51	Hexacosane	C26H54	3,853986
47,74	Hentriacontane	C31H64	1,806931
47,89	Hentriacontane	C31H64	5,449522
48,06	Tetracosanal	C24H48O	2,545504
49,03	Octacosane	C28H58	25,32232
49,47	Tetracosanoic acid, methyl ester	C25H50O2	0,764109
52,45	Nonacosane	C29H60	8,413102

Table S4. GC-MS profile of chloroform extract of *A. turkestanicum* Regel bulb (H 454 a.s.l.).

5,71	Hexanal	$C_6H_{12}O$	2,958841
9,19	Heptanal	$C_7H_{14}O$	0,322251
11,22	2-Heptenal, (E)-	$C_7H_{12}O$	0,84798
12,3	Furan, 2-pentyl-	$C_9H_{14}O$	0,225426
16,04	Nonanal	$C_9H_{18}O$	0,268657
19,1	Decanal	$C_{10}H_{20}O$	0,179415
20,6	4-Oxononanal	$C_9H_{16}O_2$	0,480131
21,73	2,4-Decadienal, (E,E)-	$C_{10}H_{16}O$	0,462261
22,37	2,4-Decadienal, (E,E)-	$C_{10}H_{16}O$	0,854615
24,31	Tetradecane	$C_{14}H_{30}$	3,160523
26,8	Pentadecane	$C_{15}H_{32}$	5,846621
29,16	Hexadecane	$C_{16}H_{34}$	7,953548
31,39	Heptadecane	$C_{17}H_{36}$	9,182275
33,5	Octadecane	$C_{18}H_{38}$	2,224402
34,5	2-Pentadecanone, 6,10,14-trimethyl-	$C_{18}H_{36}O$	6,462516
35,52	Nonadecane	$C_{19}H_{40}$	2,753287
36,11	Hexadecanoic acid, methyl ester	$C_{17}H_{34}O_2$	1,691691
37,46	Eicosane	$C_{20}H_{42}$	3,183835
39,31	Heneicosane	$C_{21}H_{44}$	6,293274
41,1	Docosane	$C_{22}H_{46}$	20,57274
42,79	Tricosane	$C_{23}H_{48}$	8,792754
45,58	Dodecanoic acid, undecyl ester	$C_{23}H_{46}O_2$	9,941228
45,99	Pentacosane	$C_{25}H_{52}$	5,341728

Table S5. Mass spectrometric characteristics of detected compounds in positive and negative electrospray ionization modes (ESI⁺/ESI⁻) of *A. suworowii* Regel bulb.

	**Negotive mode (ESI-)**					**POSITIVE MODE (ESI +)**				
	RT min	(m/z)	intensity			RT min	(m/z)	intensity		
	0.701	164.1	~6.60E5 (660 000)			0.701	136.0	~5.40E5 (540 000)		
	0.701	112.0	~4.90E5 (490 000)			0.701	684.2	~1.85E5 (185 000)		
	0.701	133.9	~4.70E5 (470 000)			0.701	120.0	~1.80E5 (180 000)		
	0.701	203.0	~4.40E5 (440 000)			0.701	149.1	~1.45E5 (145 000)		
	0.701	150.1	~3.95E5 (395 000)			0.701	227.3	~1.15E5 (115 000)		
	0.701	155.9	~2.20E5 (220 000)							
	0.701	218.0	~1.70E5 (170 000)							
	0.701	130.0	~1.40E5 (140 000)							
	0.701	232.9	~1.35E5 (135 000)							
	0.701	316.8	~1.30E5 (130 000)							
	0.467	112.0	~6.45E5 (645 000)			0.467	103.9	~3.55E6 (3 550 000)		
	0.467	169.9	~1.70E5 (170 000)			0.467	108.9	~2.75E6 (2 750 000)		
	0.467	186.1	~1.40E5 (140 000)			0.467	140.7	~2.20E6 (2 200 000)		
	0.467	352.9	~6.00E4 (60 000)			0.467	172.8	~2.15E6 (2 150 000)		
	0.467	288.9	~5.50E4 (55 000)			0.467	127.0	~1.95E6 (1 950 000)		
	0.467	255.0	~5.00E4 (50 000)			0.467	190.9	~1.70E6 (1 700 000)		
	0.467	272.9	~4.50E4 (45 000)			0.467	627.2	~1.45E6 (1 450 000)		
	0.467	113.9	~4.00E4 (40 000)			0.467	249.0	~1.15E6 (1 150 000)		
	0.467	479.6	~3.50E4 (35 000)			0.467	228.8	~1.10E6 (1 100 000)		
	0.584	196.4	~9.20E5 (920 000)			0.467	210.4	~9.50E5 (950 000)		
	0.584	439.0	~7.70E5 (770 000)			0.467	362.8	~9.00E5 (900 000)		
	0.584	111.9	~5.10E5 (510 000)			0.467	353.0	~7.00E5 (700 000)		
	0.584	155.0	~4.20E5 (420 000)			1.753	610.4	~4.90E5 (490 000)		
	0.584	296.9	~4.20E5 (420 000)			1.753	684.2	~4.15E5 (415 000)		
	0.584	316.1	~3.50E5 (350 000)			1.753	209.2	~3.10E5 (310 000)		
	0.584	250.7	~3.05E5 (305 000)			1.753	340.1	~2.95E5 (295 000)		

	0.584	358.1	~1.70E5 (170 000)			1.753	577.2	~2.20E5 (220 000)	
	0.584	217.1	~1.45E5 (145 000)			1.753	105.1	~2.05E5 (205 000)	
	0.584	232.9	~1.40E5 (140 000)			1.753	117.0	~1.70E5 (170 000)	
	0.584	574.7	~1.10E5 (110 000)			1.753	322.3	~1.45E5 (145 000)	
						1.753	611.2	~1.15E5 (115 000)	
						1.753	685.5	~1.00E5 (100 000)	
						1.753	773.6	~7.00E4 (70 000)	
	5.434	694.8	~1.43E6 (1 430 000)			5.434	610.4	~4.90E5 (490 000)	
	5.434	338.2	~8.90E5 (890 000)			5.434	684.2	~4.15E5 (415 000)	
	5.434	695.5	~5.10E5 (510 000)			5.434	209.2	~3.10E5 (310 000)	
	5.434	249.0	~4.10E5 (410 000)			5.434	340.1	~2.95E5 (295 000)	
	5.434	622.5	~2.20E5 (220 000)			5.434	340.1	~7.20E5 (720 000)	
	5.434	381.0	~1.10E5 (110 000)			5.434	362.0	~7.10E5 (710 000)	
	5.434	643.6	~8.00E4 (80 000)			5.434	227.2	~3.20E5 (320 000)	
	5.492	249.0	~9.60E5 (960 000)			5.434	114.1	~2.90E5 (290 000)	
	5.492	187.0	~9.00E4 (90 000)			5.434	577.2	~2.20E5 (220 000)	
	5.492	335.3	~8.50E4 (85 000)			5.434	105.1	~2.05E5 (205 000)	
	5.492	255.2	~7.50E4 (75 000)			5.434	117.0	~1.70E5 (170 000)	
	5.492	451.3	~6.00E4 (60 000)			5.434	322.3	~1.45E5 (145 000)	
	5.492	487.3	~5.50E4 (55 000)			5.434	611.2	~1.15E5 (115 000)	
	5.492	622.5	~5.00E4 (50 000)			5.434	685.5	~1.00E5 (100 000)	
	5.609	249.0	~7.30E5 (730 000)			5.434	773.6	~7.00E4 (70 000)	
	5.609	746.6	~2.10E5 (210 000)			5.550	453.4	~5.20E5 (520 000)	
	5.609	713.6	~1.15E5 (115 000)			5.550	475.3	~3.10E5 (310 000)	
	5.609	600.5	~9.00E4 (90 000)			5.550	627.3	~1.75E5 (175 000)	
	5.609	481.1	~8.50E4 (85 000)			5.550	340.3	~5.50E4 (55 000)	
	5.609	255.2	~5.50E4 (55 000)			5.550	435.4	~5.00E4 (50 000)	
	5.609	653.6	~5.00E4 (50 000)			5.550	773.4	~2.50E4 (25 000)	
	5.667	186.1	~6.20E5 (620 000)						
	5.667	249.0	~5.65E5 (565 000)			5.667	701.6	~3.25E5 (325 000)	

	5.667	713.7	~2.80E5 (280 000)			5.667	679.7	~2.90E5 (290 000)		
	5.667	714.6	~1.10E5 (110 000)			5.667	614.5	~1.45E5 (145 000)		
	5.667	139.0	~8.50E4 (85 000)			5.667	453.3	~8.50E4 (85 000)		
	5.667	740.6	~8.00E4 (80 000)			5.667	475.3	~7.50E4 (75 000)		
	5.667	223.2	~6.50E4 (65 000)			5.667	340.2	~7.00E4 (70 000)		
	5.667	343.2	~5.50E4 (55 000)							
	6.193	248.9	~1.03E6 (1 030 000)			6.076	475.2	~3.70E5 (370 000)		
	6.193	431.0	~8.00E5 (800 000)			6.076	487.3	~1.95E5 (195 000)		
	6.193	297.0	~4.70E5 (470 000)			6.076	610.2	~1.15E5 (115 000)		
	6.193	139.0	~3.00E5 (300 000)			6.076	684.3	~1.05E5 (105 000)		
	6.193	211.2	~1.65E5 (165 000)			6.076	363.4	~6.50E4 (65 000)		
	6.193	171.1	~1.50E5 (150 000)			6.076	758.4	~5.50E4 (55 000)		
	6.193	265.0	~1.50E5 (150 000)			6.076	149.1	~4.00E4 (40 000)		
	6.193	323.3	~1.45E5 (145 000)							
	6.193	481.1	~1.10E5 (110 000)			6.193	413.2	~4.15E5 (415 000)		
	6.193	631.2	~8.00E4 (80 000)			6.193	435.3	~2.20E5 (220 000)		
	6.252	248.9	~1.13E6 (1 130 000)			6.193	631.2	~4.50E4 (45 000)		
	6.252	297.2	~1.12E6 (1 120 000)			6.193	681.3	~4.00E4 (40 000)		
	6.252	481.1	~5.30E5 (530 000)							
	6.252	430.9	~2.50E5 (250 000)							
	6.252	311.2	~2.10E5 (210 000)							
	6.252	183.1	~1.80E5 (180 000)							
	6.252	138.3	~1.70E5 (170 000)							
	6.252	265.1	~1.30E5 (130 000)							
	6.252	118.9	~1.10E5 (110 000)							
	6.308	311.2	~2.20E5 (220 000)							
	6.308	249.0	~1.25E5 (125 000)							
	6.308	531.0	~4.00E4 (40 000)							
	6.308	481.1	~3.50E4 (35 000)							
	6.308	431.1	~2.50E4 (25 000)							

	6.308	182.9	~2.50E4 (25 000)							
	6.308	325.1	~2.00E4 (20 000)							
	6.427	311.2	~2.15E5 (215 000)							
	6.427	249.0	~1.10E5 (110 000)							
	6.427	325.2	~7.50E4 (75 000)							
	6.427	182.9	~2.20E4 (220 000)							
	6.427	531.0	~2.00E4 (20 000)							
	6.544	223.0	~1.15E5 (115 000)							
	6.544	249.0	~8.50E4 (85 000)							
	6.544	325.2	~5.20E4 (52 000)							
	6.544	339.2	~2.20E4 (22 000)							
	6.544	138.9	~2.00E4 (20 000)							

Table S6. Comparison of dominant peaks across studied *Allium* L. bulb samples

**Species**	**Modes**	**Primary Peak**		**Secondary Peak**		**Tertiary Peak**	
		**RT (min)**	**Area %**	**RT (min)**	**Area %**	**RT (min)**	**Area %**
A. suworowii	ESI(+)	0.4769	66.65%	5.4372	20.51%	5.9678	6.35%
	ESI(-)	0.4769	58.42%	6.2099	23.42%	5.9678	10.52%
A. trachyscordum	ESI(+)	0.7128	44.19%	6.0713	30.01%	5.4408	21.66%
	ESI(-)	0.7052	61.06%	6.5889	30.84%	5.4303	4.39%
A.atrosanguineum var. *fedschenkoanum*	ESI(+)	0.7051	54.84%	5.4409	24.81%	6.0251	10.45%
	ESI(-)	0.5213	50.34%	5.4383	23.34%	6.0227	17.76%
A. turkestanicum	ESI(+)	0.7216	48.59%	5.4403	26.34%	6.0879	13.03%
	ESI(-)	0.5269	49.40%	5.4391	27.88%	6.0737	15.19%
